# Clinical and molecular characteristics of invasive community-acquired *Staphylococcus aureus*infections in Chinese children

**DOI:** 10.1186/s12879-014-0582-4

**Published:** 2014-11-07

**Authors:** Yanhong Qiao, Xue Ning, Qiang Chen, Ruizhen Zhao, Wenqi Song, Yuejie Zheng, Fang Dong, Shipeng Li, Juan Li, Lijuan Wang, Ting Zeng, Yanhong Dong, Kaihu Yao, Sangjie Yu, Yonghong Yang, Xuzhuang Shen

**Affiliations:** Key Laboratory of Major Diseases in Children and National Key Discipline of Pediatrics, Ministry of Education, Beijing Pediatric Research Institute, Beijing Children’s Hospital, Capital Medical University, Beijing, P.R. China; Pediatric Department of Aviation General Hospital, Beijing, P.R. China; Jiangxi Children’s Hospital, Jiangxi, P.R. China; Shenzhen Children’s Hospital, Shenzhen, P.R. China

**Keywords:** Staphylococcus aureus, Community-acquired, Invasive infection, Child, Molecular epidemiology

## Abstract

**Background:**

This study aims to investigate the clinical features of invasive community-acquired *Staphylococcus aureus* (CA-SA) infection in Chinese children and analyze its molecular features.

**Methods:**

Clinical data and invasive CA-SA isolates were prospectively collected. Pediatric risk of mortality (PRISM) score was used for disease severity measurement. Molecular typing was then performed, followed by expression analysis for virulence genes.

**Results:**

Among 163 invasive CA-SA infection cases, 71 (43.6%) were methicillin-resistant SA (MRSA) infections and 92 (56.4%) were methicillin-susceptible SA (MSSA). A total of 105 (64.4%) children were younger than 1 year old, and 79.7% (129/163) were under 3 years age. Thirteen kinds of diseases were observed, in which bacteremia and pneumonia accounted for 65.6% (107/163) and 52.8% (86/163), respectively. A total of 112 (68.1%) patients had two or more infective sites simultaneously, and four cases (2.5%) died. CA-MSSA more frequently caused multi-sites infections, bacteremia, and musculoskeletal infection than MRSA. A total of 25 sequence types (STs) were detected. MRSA mainly comprised ST59 (49/71, 69%), whereas the most frequent clonotypes were ST88 (15/92, 16.3%), ST25 (13/92, 14.1%), ST7 (13/92, 14.1%), ST2155 (12/92, 13%), and ST188 (9/92, 9.8%) for MSSA. Seven STs were common to both MSSA and MRSA groups. No differences in clinical presentation or PRISM score were found between the two groups or among different ST. The expression levels of the four known virulence genes varied among the six main ST clones.

**Conclusions:**

Invasive CA-SA infections were characterized by high incidence and multi-site infections in young children in China. The clinical manifestations of CA-MSSA were more frequently associated with multi-site infections, bacteremia and musculoskeletal infection than those of CA-MRSA. Isolated genotypes may be relevant to the expressions of virulence genes, but not to clinical manifestations.

**Electronic supplementary material:**

The online version of this article (doi:10.1186/s12879-014-0582-4) contains supplementary material, which is available to authorized users.

## Background

*Staphylococcus aureus* (SA) causes a multitude of human infections around the world [1]. In the past decade, an increase in the community-acquired SA (CA-SA) infection rate, accompanied by a decreased incidence of hospital-acquired SA (HA-SA) infections, has been observed [2]-[4].

In 2005, Gonzalez reported 14 children with severe CA-SA infections, 13 of which experienced bacteremia and bone-joint complications simultaneously and 3 died [5]. Since 1999 four deaths caused by severe invasive community-acquired methicillin-resistant SA (CA-MRSA) were observed in America, and many serious infections caused by CA-MRSA have been reported [6]. In Cunnington’s study, eight children with serious invasive community-acquired methicillin-susceptible SA (CA-MSSA) infection were reported and their clinical manifestations were clearly described [7]. Several studies have reported invasive infections, but few of large scale studies on the clinical characteristics of invasive CA-SA infections have been performed.

As the subjective simplification of the physiological stability index, the simplified acute physiology (SAP) score is a widely used general severity scoring system used in European intensive care medicine for adult patients [8]. The pediatric risk of mortality (PRISM) score has been developed and validated in several centers in the United States in pediatric patients [9]-[11]. It was reported that the PRISM score could directly reflect the severity of the disease, and has been widely used for predicting patients’ death or survival, as reported in Portugal in 2005, the United Kingdom and Senegal in 2006, and India in 2010 [12]-[15].

Regional differences may exist in the distribution of invasive CA-MRSA isolates. For example, ST93 and ST1 are the common types in Australia [16], whereas ST8 (USA300) is common in America [17] and France [18]. In Switzerland, ST5 accounted for the majority of invasive CA-MRSA isolates [19]. Our previous study also revealed that ST59 was the dominant clone in children from the mainland of China, which was in agreement with the results reported by Taiwan [20]. However, no dominant clone has been detected yet for invasive MSSA isolates throughout the world [21],[22].

Previous studies have indicated that virulence genes may play an important role in serious SA infections [7],[22],[23]. Although various virulence genes have been reported, core genome-encoded toxins, including hemolysin-a genes (*hla*), a-type phenol-soluble modulins (*psmα*) and *RNAIII*, have been detected in almost all SA isolates [24]. Panton–Valentine leukocidin (*pvl*) was initially considered as an important virulence factor in SA; however, the role of *pvl* in the pathogenicity of SA is still under debate [25]. Compelling evidence has revealed an association between virulence gene expression and pathogenicity in animal model. For example a study in the US showed high expression levels of core genome-encoded virulence genes contributed to the high virulence of isolate USA300 in rats [26]. However, information on the relationship between virulence gene expression and clinical manifestations in patients with invasive SA infections is still lacking [27]. A correlation has also been reported between molecular type and virulence gene expression [28],[29].

In the current study, we used the PRISM III score to evaluate disease severity, described the clinical and molecular characteristics of CA-SA, and detected the expression of the *hla, psmα, RNA III* and *pvl* genes. Virulence gene expression and clinical manifestations and virulence gene expressions were then compared between invasive CA-MRSA and CA-MSSA isolates, followed by an investigation into the relationship between molecular characteristics and disease severity. The findings contribute to our understanding of the pathogenic mechanisms involved in invasive SA infections.

## Methods

### Definitions

“Community-acquired infections” are infections that are either present or incubating on admission and associated with the first positive culture result obtained within 48 h of admission [29]. Individuals with the following “high-risk” factors were excluded from the study: receiving continuous hemodialysis, receiving chemotherapy, dependent on an indwelling catheter, needing an intravenous line or percutaneous device when culturing [15].

Invasive SA refers to SA isolated from a normally sterile body site [17]. Subjects were excluded from this classification if they had positive culture results but did not show clinically relevant infective signs [17]. Staphylococcal pneumonia, necrotizing pneumonia and musculoskeletal infection were defined as described previously [20]. Patients with pneumonia were excluded if the SA isolates were recovered from the sputum. Patients with orbital infections were excluded if the SA isolates were recovered from the swabs of the eye or ear secretion only [30]. Severe pneumonia diagnosis should meet at least one of the following requirements: (1) admission to an intensive care unit; (2) necrotizing or cavitary infiltrates; and (3) empyema. Patients that did not meet one of these requirements were classified into the moderate pneumonia group [31]. Necrotizing fasciitis was diagnosed based on a surgical report, as confirmed by pathological examination [32]. Any patient who showed SA infections involving skin or soft tissue structures was categorized under skin and soft tissue infections (SSTI).

### Patients and data collection

Patients (≤14 years old) with invasive CA-SA infections were prospectively recruited from three regional Children’s hospitals between December 2011 and February 2013. Clinical data, such as general demographic information, clinical features, laboratory results, potential risk factors and treatment, were retrieved from the medical records department. PRISM III [11] was used to measure disease severity; all patients were scored based on PRISM within 24 h of admission. This study was approved by the Ethics Committees of the three Children’s hospitals in Beijing, Shenzhen and Jiangxi. A parent or guardian of each patient provided written informed consent.

### Molecular typing and screening of the key virulence genes

All isolates were sent to the Key Laboratory of Major Diseases in Beijing Children’s Hospital for SA identification*.* Only the first isolated strain from a normally sterile body site was evaluated.

Polymerase chain reaction (PCR) amplification was used for multilocus sequence typing (MLST) and staphylococcal protein A (SPA) typing. Three core genome-encoded toxin genes, including *hla*, and *psmα* and the regulator *RNAIII*, were screened by PCR [33]; *pvl* gene was also detected as described previously [33].

### Quantitative analysis of gene expression

For RNA isolation, overnight cultures were diluted 1:100 in 10 mL of tryptic soy broth (TSB) and incubated at 37°C with shaking at 180 rpm until stationary growth phase (OD_600_ ≥ 2.0). No observable differences in the growth rates were found for the SA strains in TSB. The harvested cell aliquots were pelleted by centrifugation at 12,000 rpm at −4°C for 5 min. Each pellet was washed once in an equal volume of Tris–HCl and EDTA buffer (10 mM Tris–HCl and 1 mM EDTA, pH 8), followed by re-suspension in TE buffer containing 10 g/L lysozyme and 40 mg/L lysostaphin. The mixture was then incubated at 37°C for 15 min. Total bacterial RNA was isolated using RNAiso (Takara, Japan) according to the instructions of the manufacturer. Contaminated DNA was removed by incubating the total bacterial RNA with RNase-free DNase I (30 U/100 μg of total RNA, Takara) at 37°C for 1 h. Complementary DNA (cDNA) was synthesized using the PrimeScript RT reagent kit (Takara) according to the instructions of the manufacturer. The amounts of RNA and cDNA were quantitated using a NanoDrop spectrophotometer (NanoDrop Technologies, Wilmington, DE, USA).

Real-time PCR was performed using SYBR Premix Ex Taq™ (Takara), and the primers were designed and synthesized according to a previous study [28]. The comparative Ct (2^-ΔΔCT^) method was used to quantify the expression of the genes selected, with the expression of MRSA isolate USA300 being used as a reference. Alterations in gene expression were expressed as the fold change relative to the reference; all of the reactions were performed in triplicate, and the *gyrB* gene was used as an endogenous control [33].

### Statistical analysis

Categorical variables were analyzed using the Chi-square or Fisher’s exact test. Mann–Whitney U analysis was used to compare PRISM scores between MRSA and MSSA groups. Gene expression values were normalized by log10 transformation, then analyzed by performing the Student’s t test to compare MRSA and MSSA strains. One-way analysis of variance with Dunnett’s multiple comparison was used for the PRISM score and expression level analysis among different ST or SPA types. Differences were considered statistically significant when *p* < 0.05. All analyses were performed using SPSS software (version 18.0, SPSS Inc., Chicago IL, USA).

## Results

### Demographics of the patients

A total of 163 children (99 males and 64 females; mean age, 2.4 years) were enrolled in this study. Approximately 66.3% (108/163) of the patients were less than 1 year old and 79.8% (130/163) were under 3 years old. Seventy-one patients (43.6%) suffered from MRSA infection, while 92 (56.4%) patients manifested MSSA infection. A significantly higher proportion of patients with related risk factors was observed in the MRSA group than in the MSSA group (*χ*^2^ = 11.499, df =1, *p* =0.001). No significant difference was found between the two groups in terms of age, origin, fever duration and other factors (Table 1). A total of 120 cases (73.6%) yielded PRISM scores of less than 4.0, and 20 cases (12.3%) yielded PRISM scores higher than 8.0. The Mann–Whitney U analysis revealed no significant difference in the PRISM scores between MRSA and MSSA groups (Z = −0.706, *p* = 0.48).

**Table 1:** **Demographics and clinical characteristics of patients with invasive SA infections**

Characteristics	Total No. (%)	MRSA No. (%)	MSSA No. (%)	*P*
**Patients**	163	71 (43.6)	92 (56.4)	1.0
**Gender**				
Male	99 (60.7)	37 (52.1)	62 (67.4)	0.048
Female	64 (39.3)	34 (47.9)	30 (32.6)	
Age-Median (month, IQR^a^)	6 (35.23)	8 (35.33)	6 (32.75)	0.397
<=1 mo	51 (31.3)	20 (28.1)	31 (33.7)	0.451
1 mo–1 yr	54 (33.1)	26 (36.6)	28 (30.4)	0.593
1 yr–3 yr	25 (15.3)	15 (21.2)	10 (10.9)	0.072
3 yr–14 yr	33 (20.3)	10 (14.1)	23 (25)	0.086
**Area**				
Rural	90 (55.2)	38 (53.5)	52 (56.5)	0.702
Urban	73 (44.8)	33 (46.5)	40 (43.5)	
**Risk factors** ^**b**^:	33 (20.2)	23 (32.4)	10 (10.9)	0.001
history in the past 1 yr of				
Hospitalization	21	16	5	0.001
Surgery	6	3	3	0.336
Trauma or scalds	3	1	2	0.212
Endotracheal intubation	4	3	1	1.0
Congenital heart disease	4	2	2	0.567
Other congenital diseases^c^	3	2	1	1.00
**Presenting symptoms**				
Fever	134 (82.2)	60 (84.5)	74 (80.4)	0.5
Hypothermia	4 (2.5)	2 (2.8)	2 (2.2)	1.00
Fever days-Median (IQR)	5.5 (7)	6.5 (7)	5 (6)	0.76
Shock	4 (2.5)	2 (2.8)	2 (2.2)	1.00
Coma	2 (1.2)	1 (1.4)	1 (1.1)	1.00
**Laboratory examination**				
White cell count-Median (10^9^/L IQR)	17.35 (8.47)	18.71 (9.41)	16.41 (9.52)	0.72
Neutrophil count-Median (IQR)	10.46 (7.37)	11.58 (6.44)	10.74 (7.18)	0.81
Thrombocytopenia (<100 × 10^9^/L)	12 (7.4)	5 (7)	7 (7.6)	0.89
C-reactive protein-Median (mg/L, IQR)	47.9 (19.5)	46.7 (21.3)	48.5 (19.3)	0.92
**PRISM score** Median (IQR)	2 (6)	2 (6)	2 (6)	0.48
**Hospitalization**				
hospital days-median (IQR)	15 (7)	17 (8)	14.5 (7)	0.168
Intensive care unit (ICU) admission	76 (46.6)	34 (47.9)	42 (45.7)	0.777
ICU days-Median (IQR)	9 (4)	8 (5)	9.5 (4)	0.786

Notes: MRSA: methicillin-resistant *S. aureus*; MSSA: methicillin-susceptible *S. aureus*; PRISM: pediatric risk of mortality.

^a^Interquartile range.

^b^Not mutually exclusive.

^c^One MRSA case each of congenital immunodeficiency and congenital laryngeal cyst and one MSSA case of thalassemia.

### Clinical diagnosis

Table 2 lists the disease spectrum of patients with invasive SA infection. Bacteremia (65%), pneumonia (52.8%) and musculoskeletal infection (19.6%) were the most frequent diseases. The proportion of patients with bacteremia or musculoskeletal infection caused by MSSA was significantly higher than that caused by MRSA (*χ*^2^ = 13.696, df =1, and *p* = 0.000 for bacteremia; *χ*^2^ = 5.578, df = 1, and *p* = 0.018 for musculoskeletal infection). Fifty-one cases (31.3%) were found to have single-site infections; among which, pneumonia (45.1%) and bacteremia (29.4%) were the most common symptoms. In total, 112 patients (68.7%) showed two or more infective sites simultaneously, in which 25.9% had bacteremia and pneumonia, and 19.6% had SSTI. A significantly higher proportion of patients with multi-site infections was observed in the MSSA group compared with the MRSA group (*χ*^2^ = 5.344, df =1, and *p* = 0.021). Patients with pneumonia complicated with MRSA-instigated SSTI were higher in proportion than patients with SSTI caused by MSSA (*χ*^2^ = 10.57, df =1, and *p* = 0.001). The proportion of patients displaying pneumonia alone was higher in the MRSA group than in the MSSA group (*χ*^2^ = 7.82, df = 1, and *p* = 0.005, Table 2).

**Table 2:** **Clinical characteristics of children with invasive** ***S. aureus*** **infections**

Characteristics	Total n =163 N (%)	MRSA n =71 N (%)	MSSA n = 92 N (%)	*P*
**Infection sites** ^**a**^				
Bacteremia	107 (65.6)	34 (47.9)	73 (79.3)	0.000
Pneumonia	86 (52.8)	43 (60.6)	43 (46.7)	0.08
Severe pneumonia	72 (83.7)	38 (88.4)	34 (79.1)	0.243
Necrotizing pneumonia	16 (18.6)	8 (18.6)	8 (18.6)	1.0
Moderate pneumonia	14 (16.3)	5 (11.6)	9 (20.9)	0.243
Musculoskeletal infection	32 (19.6)	8 (11.3)	24 (26.1)	0.018
Osteomyelitis	27 (84.3)	8 (100)	19 (79.2)	0.29
Arthritis	15 (46.9)	3 (37.5)	17 (70.8)	0.116
Pyomyositis	10 (31.2)	4 (50)	6 (25)	1.0
Necrotizing fasciitis	8 (25)	2 (25)	6 (25)	1.0
Meningitis	7 (3.7)	3 (4.2)	4 (4.3)	1.0
Endocarditis	3 (1.8)	2 (2.8)	1 (1.1)	0.82
Orbital abscess	4 (2.5)	4 (5.6)	0	0.073
Deep-seated abscess	4 (2.5)	1 (1.4)	3 (3.3)	0.805
Others^b^	6 (3.6)	4 (5.6)	2 (2.2)	0.457
**Clinical diagnosis**				
**Single-site infections**	51 (31.3)	29 (40.8)	22 (23.9)	0.021
Pneumonia	23 (45.1)	18 (62.1)	5 (22.7)	0.005
Bacteremia	15 (29.4)	6 (20.7)	9 (40.9)	0.117
Musculoskeletal infection	7 (13.7)	2 (6.9)	5 (22.7)	0.224
Others^c^	6 (11.8)	3 (10.3)	3 (13.6)	1.0
**Multi-site infections**	112 (68.7)	42 (59.2)	70 (76.1)	0.021
Two infection sites	87 (77.7)	35 (49.3)	52 (74.3)	0.266
Bacteremia + SSTI	22 (19.6)	10 (23.8)	12 (17.1)	0.39
Bacteremia + Pneumonia	29 (25.9)	7 (16.7)	22 (31.4)	0.084
Bacteremia-related Pneumonia	16 (14.3)	3 (7.1)	13 (18.6)	0.094
Pneumonia-related Bacteremia	13 (11.6)	4 (9.5)	9 (12.9)	0.594
Bacteremia + Musculoskeletal infection	12 (10.7)	2 (4.8)	10 (14.3)	0.207
Pneumonia + SSTI	10 (8.9)	9 (21.4)	1 (1.4)	0.001
Others^d^	14 (12.5)	7 (16.7)	7 (10)	0.302
**The infection sites ≥3**	25 (22.3)	7 (16.7)	18 (25.7)	0.266
Bacteremia + Pneumonia + SSTI	12 (10.7)	2 (4.8)	10 (14.3)	0.207
Others^e^	13 (11.6)	5 (11.9)	8 (11.4)	1.00

MRSA: methicillin-resistant *S. aureus*; MSSA: methicillin-susceptible *S. aureus*; SSTI: skin and soft tissue infections.

^a^Not mutually exclusive.

^b^One MRSA case each of lymphadenitis, peritonitis, hepatapostema, and appendiceal abscess and one MSSA case each of peritonitis and hydrocele complicated with infection.

^c^One MRSA case each of appendiceal abscess, deep-seated abscess, and orbital abscess and three MSSA cases of deep-seated abscess.

^d^Two MRSA cases of bacteremia + orbital abscess, one MRSA case each of pneumonia + musculoskeletal infection, pneumonia + meningitis, pneumonia + peritonitis, pneumonia + hepatapostema, and musculoskeletal infection + SSTI; three MSSA cases of musculoskeletal infection + SSTI; one MSSA case each of pneumonia + meningitis, bacteremia + peritonitis, endocarditis + meningitis, and bacteremia + hydrocele complicated with infection.

^e^One MRSA case each of bacteremia + meningitis + endocarditis, bacteremia + pneumonia + lymphadenitis, pneumonia + musculoskeletal infection + endocarditis, bacteremia + pneumonia + orbital abscess + meningitis, and bacteremia + pneumonia + musculoskeletal infection + SSTI; four MSSA cases of bacteremia + musculoskeletal infection + SSTI; two cases of bacteremia + pneumonia + meningitis + SSTI; one MSSA case each of bacteremia + pneumonia + musculoskeletal infection and bacteremia + pneumonia + musculoskeletal infection + SSTI.

### Treatment and follow-up

Additional file 1: Table S1 shows information relating to the medication and surgical treatment received by patients. The proportion of susceptible antibiotics used in prior empirical therapy was significantly lower in the MRSA group than in the MSSA group (*χ*^2^ = 13.49, df =1, and *p* = 0.000). No difference was observed between the MRSA and MSSA groups in the proportion of patients requiring surgical treatment (*χ*^2^ = 1.824, df =1, and *p* = 0.117).

In total, four patients died because of infection-related diseases during hospitalization; among which, three were newborn babies with bacteremia complicated with pneumonia. The remaining case suffered from MRSA bacteremia and meningitis complicated with endocarditis.

### MLST and SPA typing

A total of 25 STs were detected. MRSA strains showed 11 ST, in which 69% were ST59. Nineteen ST, including two new ST (ST2760 and ST2771), were detected in the MSSA strains. The five most dominant STs in MSSA were ST88 (15/92, 16.3%), ST25 (13/92, 14.1%), ST7 (13/92, 14.1%), ST2155 (12/92, 13%) and ST188 (9/92, 9.8%). Seven ST were observed in both MRSA and MSSA, among which the most frequent ST were ST59, ST88, ST25, ST7, ST2155 and ST188, detected in 119 strains (73%).

The MRSA strains displayed 21 SPA types, among which t437 (42/71, 59.2%) and t441 (4/71, 5.6%) were the most frequent. Forty-three SPA types, including three new types (t12861, t12862, and t16824), were found in the MSSA isolates, with the most frequent being t091 (10/92, 10.9%), t189 (8/92, 8.7%) and t078 (6/92, 6.5%). MRSA and MSSA isolates both displayed ST59-t437, ST188-t189, ST5-t002, and ST965-t062 types (see Additional file 2: Table S2).

### MLST typing and disease

**Figure 1:**
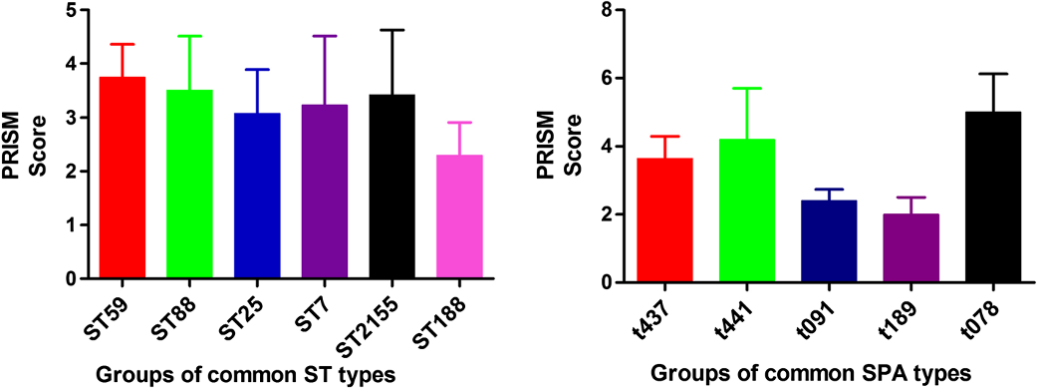
**Differences in PRISM scores between different MLST-type isolates and different Staphylococcus protein A (SPA)-type isolates.** Differences in PRISM score between different MLST-type and SPA-type groups are not statistically significant (one-way ANOVA test). Note: ST59 only had two MSSA strains. ST25, ST7, and ST2155 only comprised MSSA strains. ST188 only had one MRSA strain. ST88 had five MRSA and 15 MSSA strains. t437 only had three MSSA strains, whereas t441 only comprised MRSA strains. t091 and t189/t078 only comprised MSSA strains.

Among the 51 ST59-SA isolates, 20 strains (39.2%) led to single-site infections, including 12 (23.5%) pneumonia only, 5 (9.8%) bacteremia, and 2 (3.9%) musculoskeletal infections. A total of 31 strains (60.8%) were found to cause multi-site infections, including 8 (15.7%) bacteremia complicated with SSTI and 7 (13.7%) pneumonia complicated with SSTI. Among the 20 ST88-SA isolates, 14 (70%) resulted in multi-site infections, among which 8 cases exhibited bacteremia complicated with pneumonia or musculoskeletal infection. More than half of the ST25, ST7, ST2155 and ST188 isolates were associated with multi-site infections (Table 3). Fisher analysis showed no significant difference in the distribution of infective sites between the MRSA and MSSA strains in ST59, ST88 and ST188 type strains. No differences were observed in the infective site number and infection type (bacteremia, pneumonia, and musculoskeletal infection) among the various ST. No difference was found in the PRISM scores among the various STs and SPA types (Figure 1).

**Table 3:** **Clinical characteristics of children with invasive infections caused by the common MLST-type** ***S. aureus*** **strains**

Clinical syndrome		ST59		ST88		ST25	ST7	ST2155	ST188	
		(n = 51)		(n = 20)		(n = 13)	(n = 13)	n = 12	n = 10	
		MRSA	MSSA	MRSA	MSSA	MSSA	MSSA	MSSA	MRSA	MSSA
		(n = 49)	(n = 2)	(n = 5)	(n = 15)	(n = 13)	(n = 13)	(n = 12)	(n = 1)	(n = 9)
Single-site infection	Total	20(39.2)	0	1(5)	5(25)	4(30.8)	5(38.5)	6(50)	1(100)	2(22.2)
	Bacteremia	5(9.8)	0	0	2(10)	2(15.4)	3(23.1)	1(8.3)	1(100)	1(11.1)
	Pneumonia	12(23.5)	0	1(5)	2(10)	1(7.7)	1(7.7)	3(25)	0	0
	Musculoskeletal infection	2(3.9)	0	0	1(5)	0	1(7.7)	2(16.7)	0	1(11.1)
	Others	1^a^(2)	0	0	0	1^b^(7.7)	0	0	0	0
Multi-site infections	Total	29(56.9)	2(3.9)	4(20)	10(50)	9(69.2)	8(61.5)	6(50)	0	7(77.8)
	B + P	4(7.8)	0	2(10)	2(10)	2(15.4)	3(23.1)	1(8.3)	0	3(33.3)
	B + M	1(2)	0	1(5)	3(15)	3(23.1)	1(7.7)	2(16.7)	0	1(11.1)
	B + S	8(15.7)	0	1(5)	3(15)	2(15.4)	0	3(25)	0	1(11.1)
	P + S	7(13.7)	0	0	0	0	0	0	0	0
	B + P + S	2(3.9)	1(2)	0	1(5)	1(7.7)	1(7.7)	0	0	0
	Others	7^c^(13.7)	1^d^(2)	0	1^e^(5)	1^f^(7.7)	3^g^(23.1)	0	0	2^h^(22.2)

Notes: B + P, bacteremia + pneumonia; B + M, bacteremia + musculoskeletal infection; B + S, bacteremia + skin and soft tissue infections (SSTI); P + S, pneumonia + SSTI; B + P + S, bacteremia + pneumonia + SSTI.

^a^One case of orbital abscess.

^b^One case of deep-seated abscess.

^c^Two cases of bacteremia + orbital abscess, one case each of bacteremia + pneumonia + musculoskeletal infection, musculoskeletal infection + SSTI, bacteremia + pneumonia + lymphadenitis, bacteremia + pneumonia + osteomyelitis + SSTI, and pneumonia + osteomyelitis + endocarditis.

^d^One case of bacteremia + pneumonia + musculoskeletal infection + SSTI.

^e^One case of bacteremia + musculoskeletal infection + SSTI.

^f^One case of musculoskeletal infection + SSTI.

^g^Two cases of bacteremia + pneumonia + meningitis and one case of pneumonia + meningitis.

^h^One case each of bacteremia + pneumonia + musculoskeletal infection and meningitis + endocarditis.

### Quantitative analysis of *hla*, *psmα*, *pvl*, and *RNAIII*gene expression

PCR results revealed that all of the isolates carried *hla*, *psmα* and *RNAIII* genes. The *pvl* gene was detected in 44 strains (27%; 30 MRSA and 14 MSSA). A significantly higher ratio of isolates carrying the *pvl* gene was observed in the MRSA group than in the MSSA group (*χ*^2^ = 14.864, df =1 and *p* = 0.000). No difference was found between the MRSA and the MSSA strains in terms of *psmα*, *hla*, *RNAIII* and *pvl* expression levels (t = -0.208, df =161 and *p* = 0.835 for *psmα*; t = -0.174, df =161 and *p* = 0.862 for *hla*; t = -1.486, df =161 and *p* = 0.139 for *RNAIII*; t = 0.772, df = 161 and p = 0.441 for *pvl*; see Additional file 3: Figure S1).

**Figure 2:**
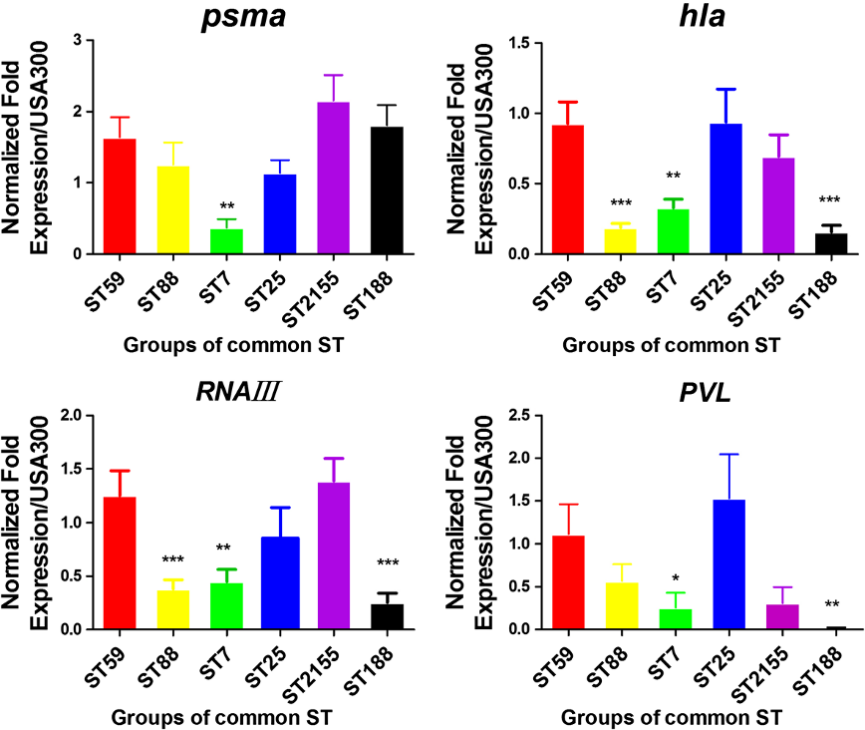
**Expression of the** ***psmα, hla, RNAIII,*** **and** ***pvl*** **genes in different MLST-type and SPA-type isolates.** The expression of key genes was measured by qRT–PCR of cultures grown to the early phase of stationary growth in TSB. gyrB cDNA was used as an endogenous control. USA300 was used as a normalized control to measure sample expression. Data was normalized by transforming the data by log10 (gene expression values). The results are the means of each group and are presented as means ± standard errors of the means. *: p <0.05, **: p <0.01, ***: p <0.001 (one-way ANOVA, Dunnett’s multiple comparison test vs. ST59).

Significant differences were observed in the gene expression levels of the four genes among the six most dominant ST genotypes (F = 3.947 and *p* = 0.003 for *psmα*; F = 7.62 and *p* = 0.00 for *hla*; F =9.276 and *p* = 0.00 for *RNAIII*; and F = 3.22 and *p* = 0.01 for *pvl*). The expression levels of the *psmα* genes in ST59 were significantly higher than those in ST7 (*p* = 0.003). Higher expression levels of *hla* and *RNAIII* were detected in the ST59 strains than those in the ST88, ST7 and ST188 strains (*p* = 0.00, *p* = 0.009 and *p* = 0.00 for *hla*; and *p* = 0.00, *p* = 0.001 and *p* = 0.00 for *RNAIII*). In addition, a higher *pvl* gene expression level was also observed in the ST59 strain than in the ST188 and ST7 strains (*p* = 0.008 and *p* = 0.021). Lower *pvl* expression was observed in ST59 than in the ST25 strains (Figure 2).

## Discussion

### Clinical characteristics of invasive CA-SA infections in children

As a pathogen with extremely high prevalence, CA-SA causes various severe clinical infections and is associated with morbidity and mortality [4],[17],[34],[35]. In Suryadevara’s study, most of the 128 children with invasive SA infections showed symptoms of bacteremia and musculoskeletal infections, and 61.8% of these children were under 4 years age [4]. Chen reported that cases in children under 1 year of age accounted for 29% of the total number of children with invasive CA-MRSA infections [36]. Klevens reported that bacteremia and pneumonia are the most common diseases in invasive MRSA infections [17]. In the present study, children younger than 1 year of age accounted for approximately 64.4% of the total cases with invasive SA infections, and bacteremia was the most common diagnosis. In addition, 68.1% of the children exhibited two or more infective sites, indicating that the clinical manifestations of invasive SA infections are complex in young children.

Previous studies have also revealed that invasive MRSA in children mainly resulted in multi-sites infections [36],[37]. However, few studies have reported the clinical features of pediatric invasive MSSA infections. In the current study, significantly higher proportion of bacteremia, musculoskeletal infection and multi-site infections were found in the pediatric MSSA group than in the MRSA group, which differed from the results reported for adults [16],[38]. Recent studies have reported that fewer MSSA cells were required to cause the same rate of death in mice than MRSA cells, indicating the higher pathogenicity of MSSA than MRSA [39]. In addition, differences in virulence of epidemic clones and sensitivities of different populations may also contribute to these results. However, no significant difference in the mortality and PRISM scores was observed between children with invasive MRSA and those with MSSA infections. In the present study, some of the cases had hospitalizations during the previous year and colonization of SA might not be ruled out among those patients. A high proportion of MRSA infections was observed in this study, and therefore the choice of effective antibiotics is important for successful management in invasive CA-SA infection.

### Molecular features of invasive CA-SA isolates

This is the first study to analyze of the molecular characteristics of invasive CA-MSSA strains isolated from children in mainland China. Diverse types including ST88, ST25, ST7, ST2155 and ST188, have been identified in this study, which differ from those detected in Europe, Australia and Taiwan [21],[22],[40]. In addition, the common clones of CA-MSSA were diverse, as reported previously in other geographical regions [21],[22],[40], indicating that MSSA strains isolated from invasive infection may also have regional characteristics.

MSSA has been proposed to gain or lose drug resistance and virulence genes through transduction of phage or Staphylococcal Cassette Chromosome mec (SCC*mec*) elements, and may finally evolve into MRSA [41]. For example, glycopeptide intermediately susceptible MRSA, discovered in Japan and in the United States, is supposedly derived from ST5-MSSA after acquiring SCCmec II [42]. In the present study, seven ST-SPA types existed in both MRSA and MSSA, and a similar mechanism may contribute to the evolution of MSSA to MRSA. However, in ST59-MSSA strains, only one strain was type t437, which was the dominant SPA type for ST59-MRSA. This finding indicates that ST59-MRSA is probably an entry clone, but did not evolve from local MSSA, similar to the results obtained by Jimenez [38].

### Molecular features and disease

Studies have reported that the virulence gene of SA may vary with ST type [22],[24],[29]. Little information exists on whether molecular types contribute to different clinical manifestations. Nair reported that invasive infections caused by USA300 and non-USA300 strains did not differ in mortality [43], whereas Wehrhahn *et al.* found that ST45-MSSA, ST47-MSSA and ST22-MRSA corresponded to bloodstream infections [22]. In this study, no significant differences were found in the PRISM scores, infective site number or infective type among different ST and SPA types. Several factors may affect such analyses, such as strain type, immune status of the host, underlying diseases, quantity of the infecting bacteria and the route of infection.

### Expression of key virulence genes

Key virulence genes***-****hla*, *psmα* and *RNAIII* may have major functions in various infections associated with SA in animal models. Li *et al.* found that the expression levels of these genes were related to disease severity in the rabbit model and proposed that the expressions of these genes can be used for assessing the virulence of MRSA [28]. Kobayashi deemed that *hla, psmα*, and *RNAIII* perform important functions in USA300-induced mouse skin infection [44]. A number of studies have shown that the pathogenic potential of MRSA may be related to the expression levels of virulence genes, especially the key virulence genes. However, differences in the expression levels of key virulence genes have not been clearly investigated between MRSA and MSSA, and among the different ST of MSSA. Our previous studies revealed that expressions of *hla*, *psma*, *RNAIII* and *pvl* in ST59 were higher than those in other MRSA STs [29]. In the present study, stronger associations were detected among the six common genotypes of the isolates and their expressions of virulence genes. However, ST was not correlated with PRISM score. Based on these results, it appears that SA virulence genes do not solely act in causing infection, they may also play a causative role by the regulation of virulence. In addition, in the present study, the *pvl* gene was only detected in 27% of the isolates, in which MRSA accounted for 68.1%. This result indicates that the *pvl* gene does not play a crucial role in SA pathogenicity.

## Conclusion

In this study, the clinical and molecular characteristics of invasive SA infections are investigated in Chinese children for the first time. Multifarious clinical manifestations were observed for invasive SA infections. MSSA may be associated with more multi-site infections, bacteremia and musculoskeletal infections. ST may contribute to different expression levels of virulence genes. The limitation of this study is that sample size is relatively low, multicenter studies are needed for further verification of our findings. However, our results help to clarify the pathogenic mechanism of this organism. These findings may have implications for rational drug use and the treatment of SA-induced invasive infections in children.

## Authors’ contributions

YHQ and XN were responsible for the study design, data collection, conducting the experiments and drafting of the manuscript; RZZ, YJZ, QC, FD and WQS were responsible for data and sample collection; LJW, JL and SPL performed the experiments; TZ and YHD performed the statistical analysis; YHY, KHY and SJY critically revised the manuscript; XZS contributed to the design of the study and drafting the manuscript, analyzing the data, and revising the final version of the manuscript. All authors approved the final manuscript.

## Additional files

## Electronic supplementary material

Additional file 1: Table S1.: Management and clinical outcomes of invasive *S. aureus* infections in hospitalized children. (DOC 53 KB)

Additional file 2: Table S2.: Molecular characteristics of strains isolated from patients with invasive *S. aureus* infections. (DOC 94 KB)

Additional file 3: Figure S1.: Expression levels of *psmα, hla, RNA III* and *pvl* in MRSA and MSSA. The expression of key genes was measured by quantitative real-time polymerase chain reaction (qRT–PCR) of cultures grown to the early phase of stationary growth in tryptic soy broth (TSB). *gyrB* cDNA was used as an endogenous control. USA300 was used as a normalized control to measure sample expression. Data was normalized by transforming the data by lg10 (gene expression values). The results are the means of every group and are presented as means±standard errors of the means. Differences in key gene expressions between the two groups are not statistically significant (Student’s *t*-test). (JPEG 750 KB)

Below are the links to the authors’ original submitted files for images.

Authors’ original file for figure 1

Authors’ original file for figure 2

Authors’ original file for figure 3

## Abbreviations

CA-SA: Community-acquired *Staphylococcus aureus*
PRISM: Pediatric risk of mortality
SA: *Staphylococcus aureus*
MRSA: Methicillin- resistant SA
HA-SA: Hospital-acquired SA
CA-MRSA: Community-acquired methicillin-resistant SA
CA-MSSA: Community-acquired methicillin-susceptible SA
*hla*: hemolysin-a genes
*psmα*: a-type phenol-soluble modulins
*pvl*: Panton–Valentine leukocidin
PCR: Polymerase chain reaction
MLST: multilocus sequence typing
STs: sequence types
SPA: staphylococcal protein A
TSB: tryptic soy broth
