# Supplementary material for: Clinical and molecular characteristics of invasive community-acquired Staphylococcus aureusinfections in Chinese children
Source: BMC Infect Dis. 2014 Nov 7;14:582. doi: 10.1186/s12879-014-0582-4 (PMC4225039; doi:10.1186/s12879-014-0582-4)
Supplement: Supplementary file 1 — Additional file 1: Table S1.: Management and clinical outcomes of invasive S. aureus infections in hospitalized children. (DOC 53 KB) [file 12879_2014_582_MOESM1_ESM.doc]

Additional file 1: Table S1. Management and clinical outcomes of invasive *S. aureus* infections in hospitalized children

| Management | Total  n = 163 | MRSA  n = 71 | MSSA  n = 92 | P value |
| --- | --- | --- | --- | --- |
| **Medical treatment** |  |  |  |  |
| Appropriate empirical antibiotic treatment | 93 (57.1) | 29 (40.8) | 64 (69.5) | 0.000 |
| Vancomycin definitive treatment with or without other antibiotics | 71 (43.6) | 54 (76.1) | 17 (18.5) | 0.000 |
| Vancomycin combination with cephalosporin or meropenem | 25 (35.2) | 16 (29.6) | 9 (52.9) | 0.143 |
| Vancomycin days-median (range) | 10 (4–38) | 11 (4–38) | 7 (4–16) | 0.001 |
| **Surgical intervention** | 55 (33.7) | 28 (39.4) | 27 (29.3) | 0.177 |
| **Pneumonia** | 86 | 43 | 43 |  |
| Surgical intervention^a^ | 22 (25.6) | 14 (32.6) | 8 (18.6) | 0.138 |
| Chest tube placement | 20 (23.3) | 13 (30.2) | 7 (16.3) | 0.126 |
| Empyema debridement | 3 (3.5) | 2 (4.7) | 1 (2.3) | 1.0 |
| Lung abscess excision | 3 (3.5) | 1 (2.3) | 2 (4.6) | 1.0 |
| Mechanical ventilation | 10 (11.5) | 6 (14) | 4 (9.3) | 0.501 |
| **Musculoskeletal infection** | 32 | 8 | 24 |  |
| Surgical intervention^a^ | 19 (59.4) | 6 (75) | 13 (54.2) | 0.42 |
| Incision and drainage | 16 (50) | 5 (62.5) | 11 (45.8) | 0.685 |
| Marrow cavity decompression by fenestration | 6 (18.8) | 1 (12.5) | 5 (20.8) | 1.0 |
| Arthrotomy | 5 (15.6) | 1 (12.5) | 4 (16.7) | 1.0 |
| Joint dislocation traction | 3 (9.4) | 2 (25) | 1 (4.2) | 0.147 |
| **Orbital abscess** | 4 | 4 | 0 | 0.073 |
| Orbitotomy | 4 (100) | 4 (100) | 0 | -- |
| **Prognosis** |  |  |  |  |
| Infection-related hospital deaths | 4 (2.5%) | 2 (2.8%) | 2 (2.2%) | 1.0 |

a. Not mutually exclusive
