# Supplementary material for: Clinical and molecular characteristics of invasive community-acquired Staphylococcus aureusinfections in Chinese children
Source: BMC Infect Dis. 2014 Nov 7;14:582. doi: 10.1186/s12879-014-0582-4 (PMC4225039; doi:10.1186/s12879-014-0582-4)
Supplement: Supplementary file 2 — Additional file 2: Table S2.: Molecular characteristics of strains isolated from patients with invasive S. aureus infections. (DOC 94 KB) [file 12879_2014_582_MOESM2_ESM.doc]

Additional file 2: Table S2. Molecular characteristics of strains isolated from patients with invasive *S. aureus* infections

| Isolates | Total  n = 163 |  | MRSA  n = 71 | | |  | | MSSA  n = 92 | | |
| --- | --- | --- | --- | --- | --- | --- | --- | --- | --- | --- |
| MLST | No. (%) | No. (%) | | Spa type | No. | | No.(%) | | Spa type | No. |
| ST59 | 51 (31.3) | 49 (69) | | t437 | 38 | | 2 (2.2) | | t437 | 1 |
|  |  |  |  | t441 | 4 | |  |  | t7013 | 1 |
|  |  |  |  | t163 | 1 | |  |  |  |  |
|  |  |  |  | t519 | 1 | |  |  |  |  |
|  |  |  |  | t3523 | 2 | |  |  |  |  |
|  |  |  |  | t3485 | 1 | |  |  |  |  |
|  |  |  |  | t7637 | 1 | |  |  |  |  |
|  |  |  |  | t7496 | 1 | |  |  |  |  |
| ST88 | 20 (12.3) | 5 (7) | | t186 | 2 | | 15 (16.3) | | t1376 | 5 |
|  |  |  |  | t7637 | 1 | |  |  | t2592 | 3 |
|  |  |  |  | t8723 | 1 | |  |  | t5351 | 2 |
|  |  |  |  | t4431 | 1 | |  |  | t8296 | 2 |
|  |  |  |  |  |  | |  |  | t12864 | 2 |
|  |  |  |  |  |  | |  |  | t4333 | 1 |
| ST25 | 13 (8) | 0 | |  |  | | 13 (14.1) | | t078 | 6 |
|  |  |  |  |  |  |  |  |  | t349 | 4 |
|  |  |  |  |  |  |  |  |  | t081 | 2 |
|  |  |  |  |  |  |  |  |  | t287 | 1 |
| ST7 | 13 (8) | 0 | |  |  | | 13 (14.1) | | t091 | 10 |
|  |  |  |  |  |  |  |  |  | t1743 | 2 |
|  |  |  |  |  |  |  |  |  | t796 | 1 |
| ST2155 | 12 (7.4) | 0 | |  |  | | 12 (13) | | t8822 | 5 |
|  |  |  |  |  |  |  |  |  | t9518 | 2 |
|  |  |  |  |  |  |  |  |  | t7002 t20911 t2087 t8692 t12861 | 1/each |
| ST188 | 10 (6.1) | 1 (1.4) | | t189 | 1 | | 9 (9.8) | | t189 | 8 |
|  |  |  |  |  |  |  |  |  | t8807 | 1 |
| ST5 | 6 (3.7) | 2 (2.8) | | t002 | 1 | | 4 (4.3) | | t002 t179 t548 t954 | 1/each |
|  |  |  |  | t601 | 1 | |  |  |  |  |
| ST6 | 6 (3.7) | 0 | |  |  | | 6 (6.5) | | t701 | 3 |
|  |  |  |  |  |  |  |  |  | t121 t304 t377 | 1/each |
| ST338 | 5 (3.1) | 5 (7) | | t437 | 4 | | 0 | |  |  |
|  |  |  |  | t1751 | 1 | |  |  |  |  |
| ST15 | 4 (2.5) | 0 | |  |  | | 4 (4.3) | | t9531 | 2 |
|  |  |  |  |  |  |  |  |  | t084 | 2 |
| ST239 | 3 (1.) | 3 (4.2) | | t037 t030 t2270 | 1/each | | 0 | |  |  |
| ST965 | 3 (1.8) | 2 (2.8) | | t062 | 2 | | 1 (1.1) | | t062 | 1 |
| ST630 | 2 (1.2) | 1 (1.4) | | t4549 | 1 | | 1 (1.1) | | t349 | 1 |
| ST30 | 2 (1.2) | 0 | |  |  | | 2 (2.2) | | t338 t4471 | 1/each |
| ST72 | 2 (1.2) | 1 (1.4) | | t664 | 1 | | 1 (1.1) | | t148 | 1 |
| ST375 | 1 (0.6) | 1 (1.4) | | t2270 | 1 | | 0 | |  |  |
| ST1777 | 1 (0.6) | 1 (1.4) | | t318 | 1 | | 0 | |  |  |
| ST121 | 1 (0.6) | 0 | |  |  | | 1 (1.1) | | t9518 | 1 |
| ST398 | 1 (0.6) | 0 | |  |  | | 1 (1.1) | | t12862 | 1 |
| ST509 | 1 (0.6) | 0 | |  |  | | 1 (1.1) | | t375 | 1 |
| ST1301 | 1 (0.6) | 0 | |  |  | | 1 (1.1) | | t3666 | 1 |
| ST1921 | 1 (0.6) | 0 | |  |  | | 1 (1.1) | | t164 | 1 |
| ST2196 | 1 (0.6) | 0 | |  |  | | 1 (1.1) | | t7960 | 1 |
| ST2771 | 2 | 0 | |  |  | | 2 | | t437 | 2 |
| ST2760 | 1 | 0 | |  |  | | 1 | | t189 | 1 |
