# Supplementary figures and images for: Clinical and molecular characteristics of invasive community-acquired Staphylococcus aureusinfections in Chinese children
Source: BMC Infect Dis. 2014 Nov 7;14:582. doi: 10.1186/s12879-014-0582-4 (PMC4225039; doi:10.1186/s12879-014-0582-4)

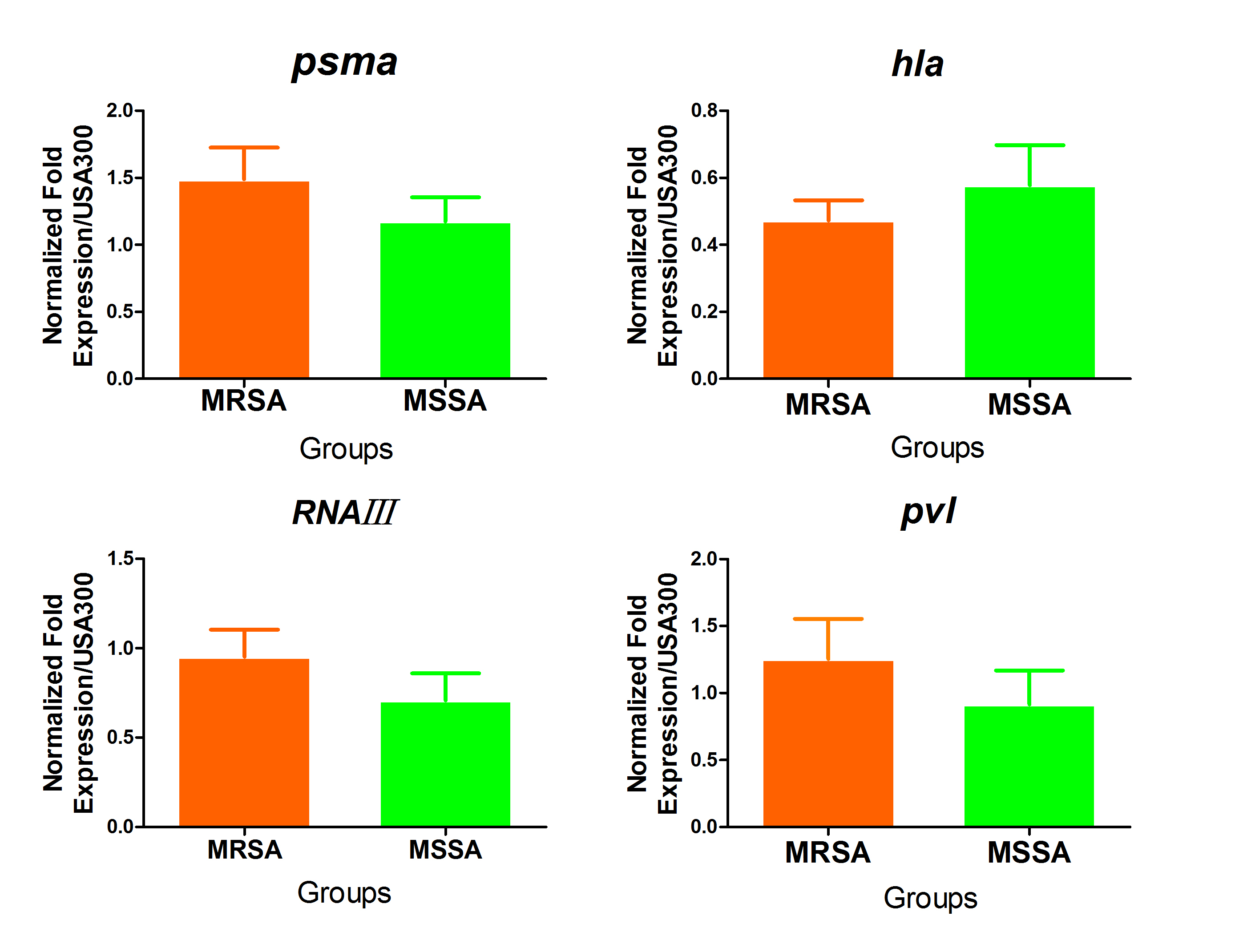

Supplement: Supplementary file 3 — Additional file 3: Figure S1.: Expression levels of psmα, hla, RNA III and pvl in MRSA and MSSA. The expression of key genes was measured by quantitative real-time polymerase chain reaction (qRT–PCR) of cultures grown to the early phase of stationary growth in tryptic soy broth (TSB). gyrB cDNA was used as an endogenous control. USA300 was used as a normalized control to measure sample expression. Data was normalized by transforming the data by lg10 (gene expression values). The results are the means of every group and are presented as means±standard errors of the means. Differences in key gene expressions between the two groups are not statistically significant (Student’s t-test). (JPEG 750 KB) [file 12879_2014_582_MOESM3_ESM.jpeg]

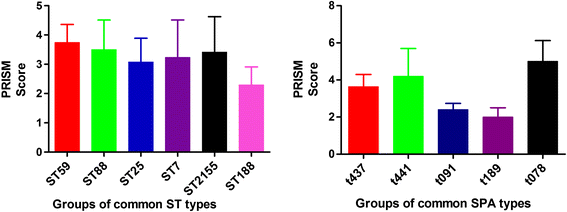

Supplement: Supplementary file 4 — Authors’ original file for figure 1 [file 12879_2014_582_MOESM4_ESM.gif]

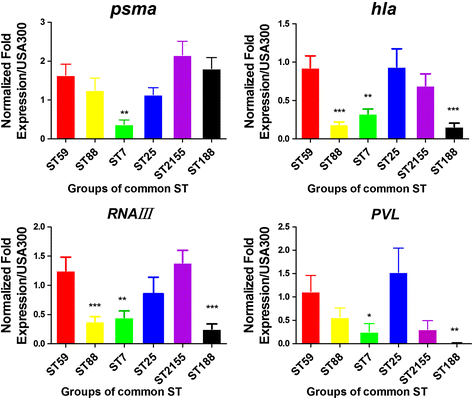

Supplement: Supplementary file 5 — Authors’ original file for figure 2 [file 12879_2014_582_MOESM5_ESM.gif]

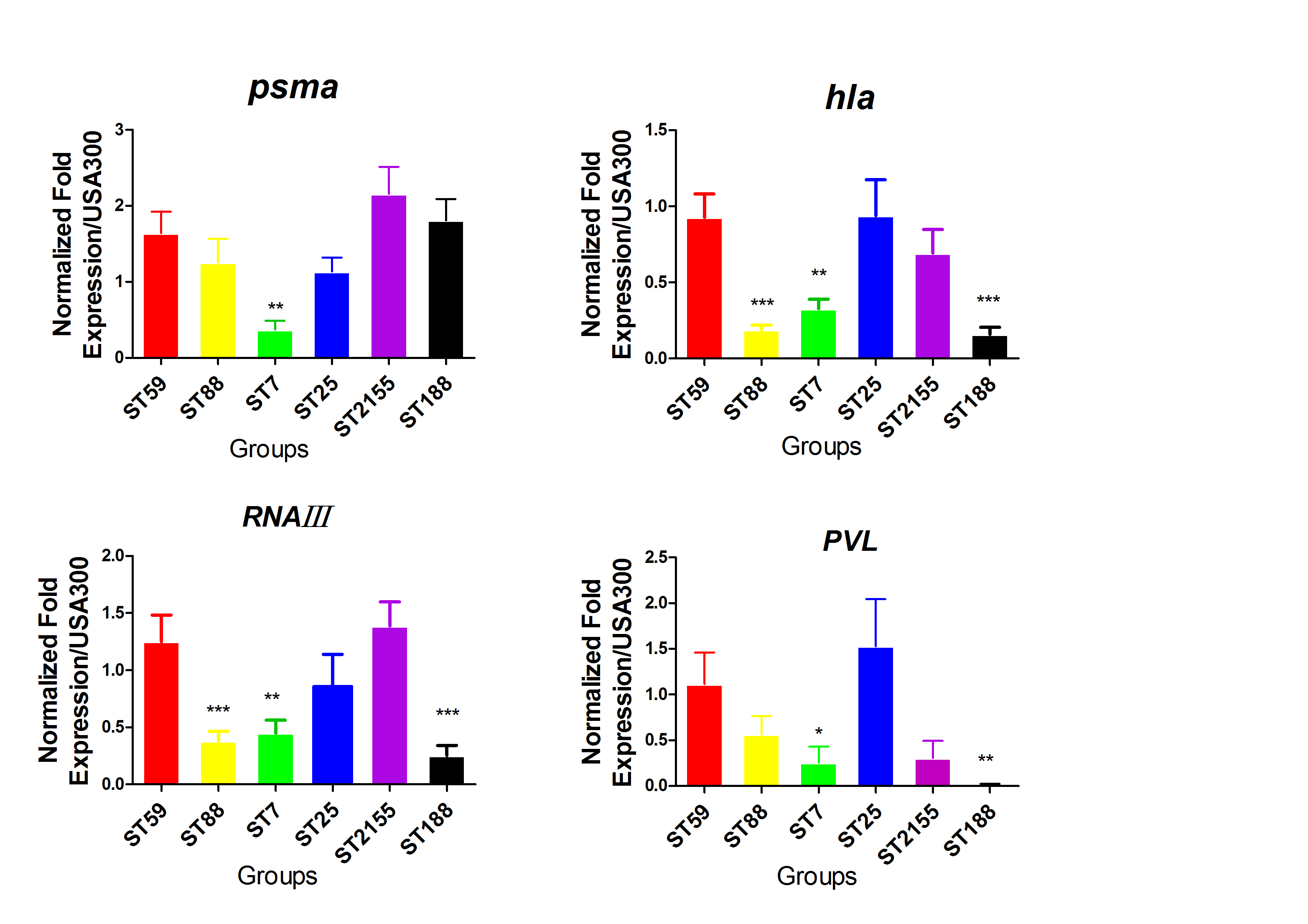

Supplement: Supplementary file 6 — Authors’ original file for figure 3 [file 12879_2014_582_MOESM6_ESM.jpeg]
